# Metastatic Leiomyoma Following Menopause: A Case Report and Review of Literature

**DOI:** 10.7759/cureus.31549

**Published:** 2022-11-15

**Authors:** Shoko Otsuka, Shintaro Yanazume, Mika Mizuno, Shinichi Togami, Hiroaki Kobayashi

**Affiliations:** 1 Department of Obstetrics and Gynecology, Kagoshima University Hospital, Kagoshima, JPN

**Keywords:** menopause, lung metastasis, hormone replacement therapy, parasitic, metastasizing leiomyoma

## Abstract

Uterine leiomyomas commonly reduce naturally after menopause. We report a rare case of metastasizing leiomyoma that grew after surgical menopause. A 68-year-old woman suffered from pelvic and lung masses without clinical symptoms. Nineteen years ago, she underwent a total hysterectomy and bilateral adnexectomy for multiple uterine myomas and bilateral endometriotic cysts. She has since been regularly prescribed conjugated estrogens. Surgery was scheduled in order to rule out malignancy; abdominal masses resection and thoracoscopic left partial pulmonary resection (S3, S4, S10) were performed. The histological diagnosis was leiomyoma in both abdominal and lung masses, and there was no evidence of gene mutations, which suggested that leiomyosarcoma was indicated. This case may indicate that hormone replacement was augmented via derived nutrient vessels after a surgical ovarian absence.

## Introduction

Uterine leiomyomas are the most common benign tumors in gynecology. Most uterine leiomyomas are commonly detected through changes in the expression of estrogen receptors (ER) and progesterone receptors (PR), and these female hormones are involved in proliferation. Uterine leiomyomas gradually reduce naturally after menopause. Metastasizing leiomyoma (ML) has been known as a rare phenomenon of uterine leiomyoma extending outside the uterus, and it often requires a differential diagnosis from the metastasis of malignant tumors.

Here, we report a very rare case of ML that grew in the pelvic cavity and the lungs during long-term hormone replacement therapy (HRT) after surgical menopause following a hysterectomy.

## Case presentation

A 68-year-old, multiparous woman was referred to our hospital for treatment of multiple abdominal masses with no clinical symptoms. This patient had normal stature (154 cm), with a body weight of 56 kg and had a history of micro-cerebral infarction. At 49 years old, she underwent a total hysterectomy and bilateral adnexectomy for multiple uterine myomas and bilateral endometriotic cysts associated with menorrhagia. Then she was regularly prescribed conjugated estrogens (0.625 mg once every three days) administered in the gynecologic clinic for surgical menopause. She underwent trans-vaginal ultrasonography regularly during the oral administration. At the age of 64, a tumor with a major diameter of 4 cm was found at the right vaginal stump, while there were no clinical symptoms such as abdominal pain, ileus or respiratory discomfort. This mass gradually enlarged and a new mass with a 10 cm diameter was also present, so she was considered to be treated with surgery. The patient had been taking conjugated estrogen preparations until one month before being referred to our hospital.

**Figure 1:**
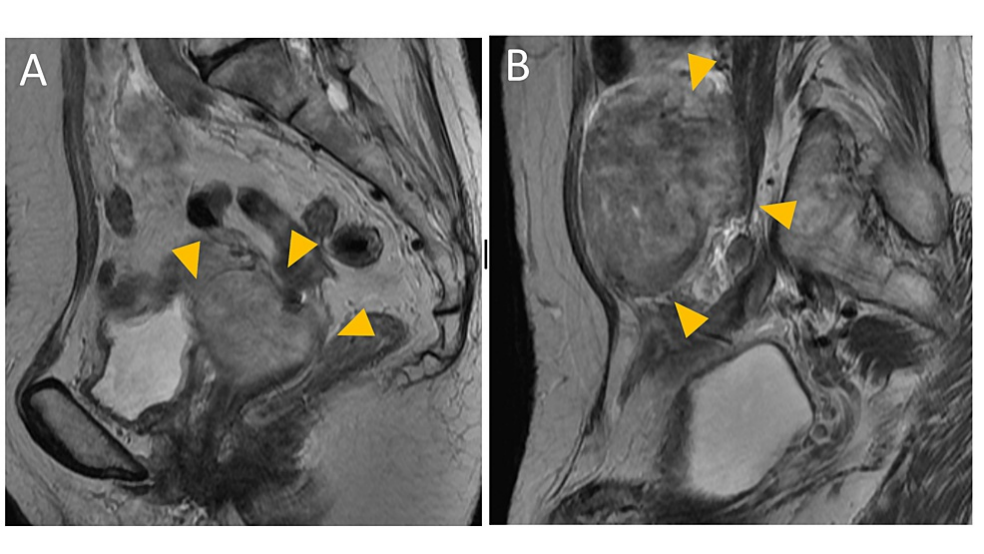
T2-weighted sagittal magnetic resonance imaging (A) Pelvic mass above the vaginal cuff (arrowheads). (B) Abdominal mass under the umbilicus (arrowheads). On the right side of the vaginal cuff, a nodule with a long diameter of 55 mm was found, with slight high intensity and relatively uniform enhancement on T2-weighted images. On the left side of the pelvis, there was a well-demarcated, solid mass lesion with a long axis of about 10 cm, showing slight high intensity on T2-weighted images. The contrast enhancement was similar to that of the vaginal cuff nodule, and on diffusion-coordinated imaging, both masses were hyperintense, but without a clear tendency to invade surrounding structures. Enlargement of residual uterine myomas and metastatic myomas were suspected.

T2-weighted sagittal magnetic resonance imaging (MRI) demonstrated 5 cm and 10 cm diameter irregular solid tumors present over the vaginal cuff extending to the right pelvic cavity and under the umbilicus of the abdominal cavity (Figure 1).

**Figure 2:**
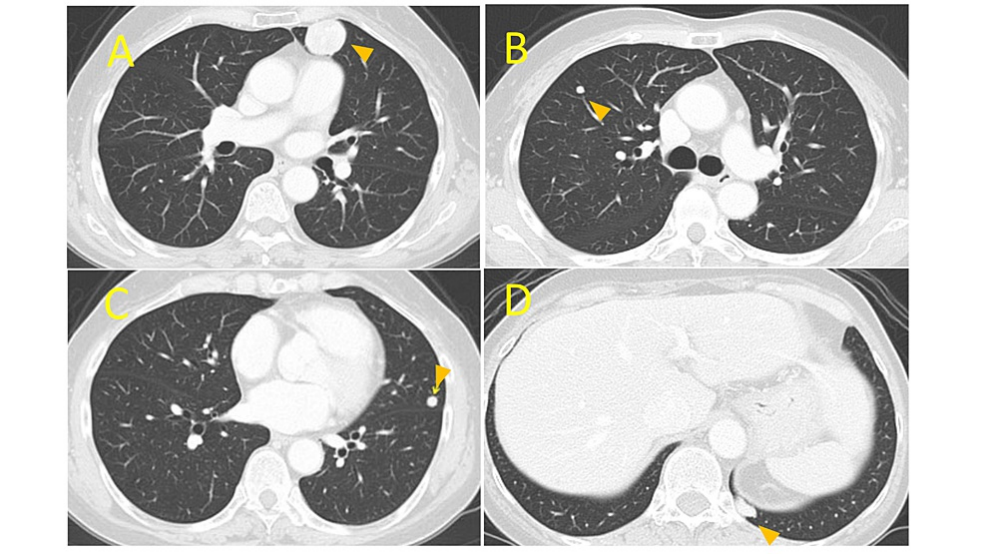
Multiple lung metastases in computed tomography Different-sized multiple lung metastases were indicated bilaterally. (A) Left upper lung S3 (arrowhead): metastatic tumor with 5 cm diameter. (B) Left lower lung S10 (arrowhead): metastatic tumor with 1 cm diameter. (C) Right upper lung S3 (arrowhead): metastatic tumor with 1 cm diameter. (D) Left lingula lung* S5* (arrowhead): metastatic tumor with 2 cm diameter.

The solid components of the tumors showed mixed-intensity signals in both T1 and T2-weighted MRI scans, and no clear invasion to the surrounding organs was suspected, suggesting a diagnosis of leiomyoma. Computed tomography (CT) indicated multiple solid masses of 5-15 mm diameter of clear demarche in the bilateral lung suspected to be ML (Figure 2).

Positron emission tomography/computed tomography (PET/CT) images showed a slight accumulation of standardized uptake value (SUV) max (1.61-1.88) present in the pelvic masses while no accumulation was present in the lung masses. None of the serum tumor markers regarding gynecological malignancies had increased.

**Figure 3:**
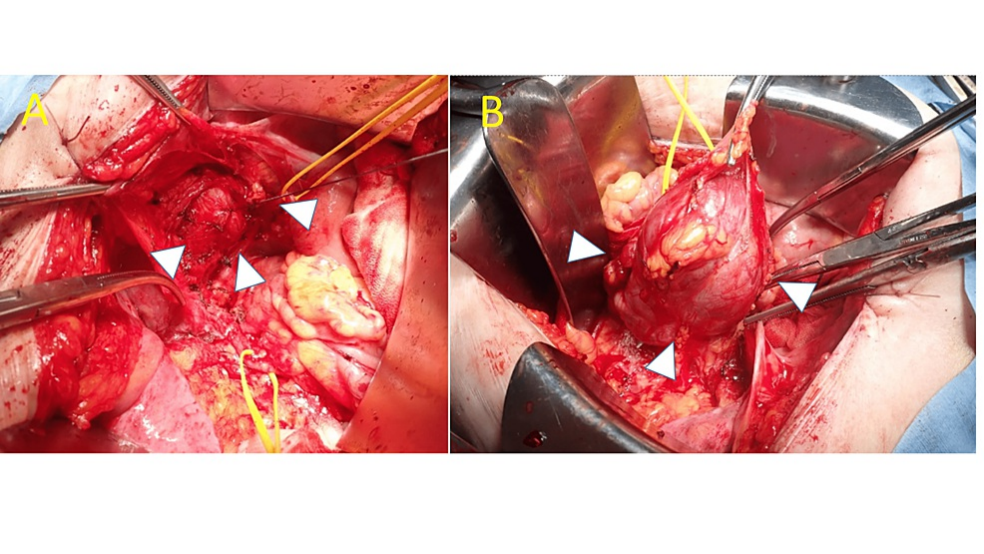
Intraoperative findings of the laparotomy (A) Pelvic mass above the vaginal cuff (arrowheads). (B) Abdominal mass under the umbilicus (arrowheads).

Surgery for the abdominal masses was scheduled in order to rule out malignancy with abdominal mass and vaginal cuff resection being performed. No lesions other than these two masses were found in the abdominal cavity (Figure 3).

**Figure 4:**
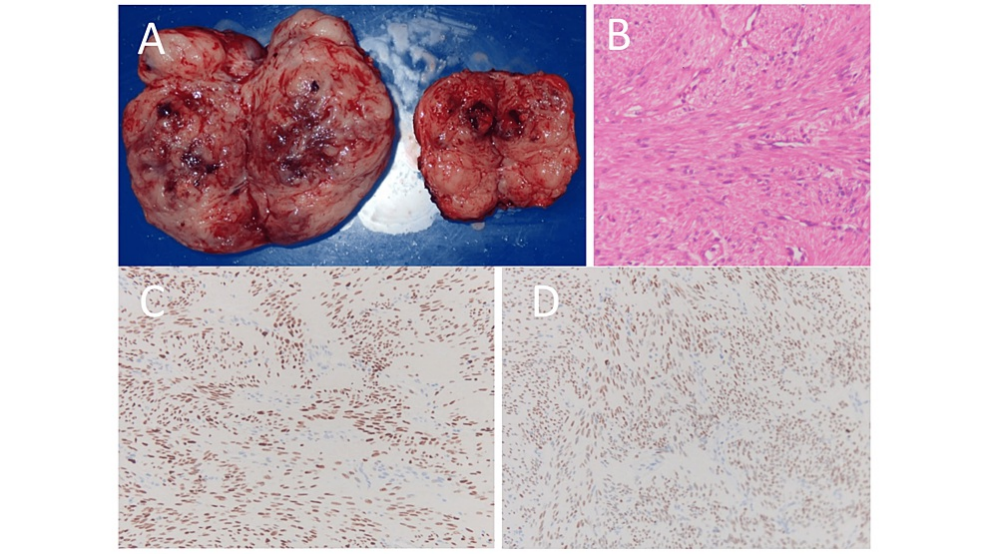
Resected tumors and histopathological findings (A) Left: abdominal mass; Right: Pelvic mass above the vaginal cuff. The tumor just above the vaginal cuff measured 5×5×3 cm, and the tumor growing in the left abdominal cavity was 9×7×3 cm. The cross-section was grayish-white. The resected lung also showed a well-demarcated, solid mass with a grayish-white cross-section. (B) Spindle cells with abdominal mass, hematoxylin-eosin (HE) ×200. (C-D) Immunohistochemical findings of the abdominal mass (×200). Estrogen receptor (C) and progesterone receptor (D) were positive in most spindle tumor cells (×200).

The results of the postoperative histopathological examination diagnosed leiomyomas (Figure 4A-4B). Two months later, thoracoscopic left partial pulmonary resection (S3, S4, S10) was performed to rule out malignancy. Histopathological diagnosis of lung masses was leiomyoma similar to peritoneal leiomyoma, and ML was finally diagnosed. Immunohistopathologically, spindle cells were positive for alpha smooth muscle actin (α-SMA) and desmin, negative for CD10, S-100, STAT6, and CD34, and the Ki67-1 positive rate was about 1%, consistent with benign ML. ER and PR were positive in most spindle tumor cells (Figure 4C-4D).

Our custom panel next-generation sequencing (NGS) analysis using the Gynecologic Cancer Panel Version 2 [1] and an Illumina MiSeq® system (Illumina, Inc., San Diego, USA) revealed no remarkable gene mutations in the pelvic mass. A plain chest CT was performed four months after the operation, and no new lesions appeared.

## Discussion

Following long-term HRT after the patient underwent a surgically resected uterus and bilateral adnexectomy for multiple uterine myoma leading to surgical menopause, massive ML developed not only in the abdominal cavity but also the bilateral lung.

In related literature, extrauterine leiomyomas regardless of surgical interventions were so-called parasitic myomas (PM) or ML. However, extrauterine lesions other than intravenous leiomyomatosis are classified as ML according to the World Health Organization (WHO) classification fifth edition.

However, PM was included in the International Federation of Gynecology and Obstetrics (FIGO) classification of uterine myomas and is considered to be uterine myomas that exist apart from the uterus and are governed by a blood flow separate from the uterine artery. Regarding the mechanism of development, fragments generated when myomas were cut into small pieces during surgery possibly remained in the patient and grew slowly. Many patients have symptoms such as abdominal pain, pelvic pain, coital pain, abdominal distension, frequent urination, and constipation, while it has been reported that about 20% are asymptomatic [2].

ML is defined as the condition in which women have extrauterine metastases of leiomyoma with a history of uterine leiomyoma. The most frequent surgical procedure is a total hysterectomy for uterine myoma, which is often found before menopause. The most common site of metastasis is the lung, and about half of these patients experienced multiple lesions. The major symptoms of lung metastasis include cough, dyspnea, shortness of breath, and chest pain, and about 40% of them are discovered during follow-up examinations [3].

There are several theories about the cause of ML: 1) hormone-sensitive smooth muscle bundles originated in the lung grew by exposure to sex hormones and 2) the metastasis of very low-grade uterine leiomyosarcoma [4,5]. ML may also include lung metastases of undiagnosed intravenous leiomyomatosis [5]. In addition, ER and PR are positive by immunostaining, and spontaneous regression generally occurs due to ovarian castration [6]. Although standard treatments for PM and ML have not been established, reported treatment includes surgical resection, hormone therapy by progesterone for unresectable cases, and pseudo-menopausal therapy using gonadotropin-releasing hormone (GnRH) agonists [7].

In this case, the histological findings of the tumors resected in the pelvis were similar to the resected lung tumors. The proliferative index was consistent with uterine leiomyoma. The decision of surgical resections in pelvic or lung masses is important for distinguishing ML from uterine leiomyosarcoma. The tumors were suggestive of metastases in the lungs, clarifying the pathological diagnosis by surgical resection rather than hormone therapy. The result of positive for ER and PR supports the hypothesis of residual lesions after surgery gradually increasing due to long-term HRT. An important point in this case is the continuous administration of HRT for a long duration of approximately 20 years and the advanced age of the patient.

Table 1 shows a review of 11 cases of postmenopausal PM and ML found in 10 publications [8-17]

**Table 1:** A review of postmenopausal parasitic myoma and metastatic leiomyoma A review of 11 cases of postmenopausal PM and ML found in 10 publications [8-17]. SMA, smooth muscle actin; HMB, beta-hydroxy-beta-methylbutyrate; ER, estrogen receptor; PR, progesterone receptor; CD, clusters of differentiation; CK, cytokeratin; STAT, signal transducer and activator of transcription; PM, parasitic myoma; ML, metastasizing leiomyoma.

Author, date of report	Diagnostic opportunity	History of uterine myoma	Past gynecological surgery	Age of onset	Time since hysterectomy (years)	Location	Maximum tumor diameter (cm)	Postmenopausal hormone therapy	Pathological findings	Treatment	Prognosis
Boavida Ferreira et al. 2022 [8]											
Case 1	abdominal pain	unknown	－	61	－	pelvis	18.5	－	Leiomyoma, with intravenous leiomyomatoses	surgical resection	cure
Case 2	abdominal pain, distention	+	hysterectomy+Bilateral salpingo-oophorectomy	25	5	pelvis, peritoneum	25	－	Leiomyoma, SMA(+), desmin(+)	surgical resection + hormone therapy	more than once of recurrence
Marasioni et al. 2021 [9]	genital bleeding	+	－	57	－	sigmoid colon lumen	2.8	－	Leiomyoma	surgical resection	cure
Ho et al. 2020 [10]	regular follow-up	+	hysterectomy	60	25	vaginal cuff	7.2	－	Leiomyoma, SMA(+), vimentin(+)	surgical resection	cure
Patel et al. 2022 [11]	regular follow-up	+	hysterectomy+right salpingo-oophorectomy	59	15	vaginal wall to peritoneum	8.2	+	Leiomyoma	surgical resection	cure
Barik et al. 2022 [12]	low abdominal pain, distention	－	－	75	－	pelvis	10.7	－	Leiomyoma	surgical resection	cure
Osegi et al. 2019 [13]	abdominal distention	+	－	58	－	greater omentum	25	－	Leiomyoma with cystic change	surgical resection	cure
Mauduit et al. 2018 [14]	regular follow-up	+	hysterectomy	70	47	lung	2	－	Leiomyoma, HMB45(-), Ki67<1%	surgical resection	cure
Efared et al. 2017 [15]	chronic cough	+	hysterectomy	57	8	lung	8	－	Leiomyoma ER(＋), PR(+), SMA(+), desmin(+), CD34(-), S-100(-), Ki67<5%	hormone therapy	cure
Ponea et al. 2013 [16]	orthopnea, right-sided back pain	+	－	62	－	lung	3	－	Leiomyoma SMA(+), desmin(+), vimentin(+), CK7(+)	surgical resection	cure
Funakoshi et al. 2004 [17]	regular follow-up	+	hysterectomy+Bilateral salpingo-oophorectomy	77	12	lung	3.5	－	Leiomyoma, ER(-)	surgical resection	cure
This case 2022	regular follow-up	+	hysterectomy+Bilateral salpingo-oophorectomy	68	19	pelvis, lung	9	+	Leiomyoma, ER(＋), PR(+), SMA(+), desmin(+), CD10(-), CD34(-), S-100(-), STAT6(-), Ki67<1%	surgical resection	almost cure

Nine out of the 11 cases had a clear history of uterine myoma [8-11,13-17] and six out of the 10 had a history of total hysterectomy [8,10,11,14,15,17], which may include cases in which remnants of the residual tumor were discovered after menopause. On the other hand, there were three postmenopausal patients who underwent HRT, including a case in our department, and all of them tended to increase during HRT [11]. However, there have been no cases of complications with pulmonary metastasis during HRT. Interestingly, seven cases were found to be symptomatic [8,9,12,13,15,16], and none of the patients was receiving HRT. Usually, enlargement of uterine leiomyoma that was generally estrogen-dependent after menopause is rare, but these symptoms may have appeared due to postmenopausal enlargement. It is hypothesized that the mechanism leading to enlargement is the production of a small amount of estrogen derived from the ovaries, or the influence of steroid hormones other than the ovaries, and factors other than hormones. The two patients undergoing HRT, including this case [11], had no symptoms or lesions detected during regular follow-ups at the gynecological department. The guidelines revealed that the management of HRT, including the duration of replacement, or uniform age that limits the continuation of HRT, is not an issue. However, the risk assessment should be done regularly and a regular check is recommended at least annually.

In our patient, the tumor was discovered before respiratory symptoms or abdominal distention because the patient received regular follow-ups from the previous doctor who was administering HRT. In our gene panel tests, which were focused on rare malignant tumors and rapidly progressing malignant tumors, we also examined the evaluation of clonality between pelvic and lung tumors, while the efficacy could not be achieved.

In recent years, the search for genetic abnormalities in benign ML has advanced, and a comparison with genetic abnormalities in leiomyosarcoma has also been conducted [18]. The most common genome variants in leiomyosarcomas include abnormalities in TP53, RB1, PTEN, MED12, YWHAE, and VIPR2 [19]. In our custom panel, TP53, RB1, PTEN, and MED12 could be analyzed, and samples of abdominal and lung tumors in our patient did not detect any mutations. These findings were consistent with leiomyoma.

Other methods to clinically differentiate between leiomyosarcoma and ML, such as changes in microRNA (miRNA) expression, have been noted. It has been reported that its expression pattern changes in various malignant tumors, and it is expected as a biomarker for the diagnosis and treatment of a wide range of diseases [18]. It has been revealed that the expression of miRNA221 is enhanced in various malignant tumors that are commonly detected in uterine leiomyosarcoma, while not expressed in ML [20]. It is expected that it will be applied to clinical diagnosis in the future.

## Conclusions

In conclusion, this patient's case demonstrates that the progression of PM or ML via derived nutrient vessels should be considered when the patient has undergone HRT following hysterectomy and bilateral adnexectomy for uterine myoma. ML should be ruled out by pelvic examination or imaging if the patient has abdominal or respiratory masses of unknown origin.
